# Localization-enhanced moiré exciton in twisted transition metal dichalcogenide heterotrilayer superlattices

**DOI:** 10.1038/s41377-023-01171-w

**Published:** 2023-05-12

**Authors:** Haihong Zheng, Biao Wu, Shaofei Li, Junnan Ding, Jun He, Zongwen Liu, Chang-Tian Wang, Jian-Tao Wang, Anlian Pan, Yanping Liu

**Affiliations:** 1 School of Physics and Electronics, Hunan Key Laboratory for Super-microstructure and Ultrafast Process, Central South University, 932 South Lushan Road, 410083 Changsha, Hunan China; 2 State Key Laboratory of High-Performance Complex Manufacturing, Central South University, 932 South Lushan Road, 410083 Changsha, Hunan China; 3 School of Chemical and Biomolecular Engineering, The University of Sydney, Sydney, NSW 2006 Australia; 4 The University of Sydney Nano Institute, The University of Sydney, Sydney, NSW 2006 Australia; 5 Beijing National Laboratory for Condensed Matter Physics, Institute of Physics, Chinese Academy of Sciences, 100190 Beijing, China; 6 School of Physical Sciences, University of Chinese Academy of Sciences, 100049 Beijing, China; 7 Songshan Lake Materials Laboratory, 523808 Dongguan, Guangdong China; 8 Hunan Institute of Optoelectronic Integration, College of Materials Science and Engineering, Hunan University, 410082 Changsha, Hunan China; 9 Shenzhen Research Institute of Central South University, 518000 Shenzhen, China

**Keywords:** Single photons and quantum effects, Photonic devices

## Abstract

The stacking of twisted two-dimensional (2D) layered materials has led to the creation of moiré superlattices, which have become a new platform for the study of quantum optics. The strong coupling of moiré superlattices can result in flat minibands that boost electronic interactions and generate interesting strongly correlated states, including unconventional superconductivity, Mott insulating states, and moiré excitons. However, the impact of adjusting and localizing moiré excitons in Van der Waals heterostructures has yet to be explored experimentally. Here, we present experimental evidence of the localization-enhanced moiré excitons in the twisted WSe_2_/WS_2_/WSe_2_ heterotrilayer with type-II band alignments. At low temperatures, we observed multiple excitons splitting in the twisted WSe_2_/WS_2_/WSe_2_ heterotrilayer, which is manifested as multiple sharp emission lines, in stark contrast to the moiré excitonic behavior of the twisted WSe_2_/WS_2_ heterobilayer (which has a linewidth 4 times wider). This is due to the enhancement of the two moiré potentials in the twisted heterotrilayer, enabling highly localized moiré excitons at the interface. The confinement effect of moiré potential on moiré excitons is further demonstrated by changes in temperature, laser power, and valley polarization. Our findings offer a new approach for localizing moiré excitons in twist-angle heterostructures, which has the potential for the development of coherent quantum light emitters.

## Abstract (web summary)

Localized moiré excitons were found in twisted heterotrilayer superlattices, with the moiré potential depth tunable through layer degrees of freedom. This discovery benefits quantum light emitter development.

## Graphical abstract

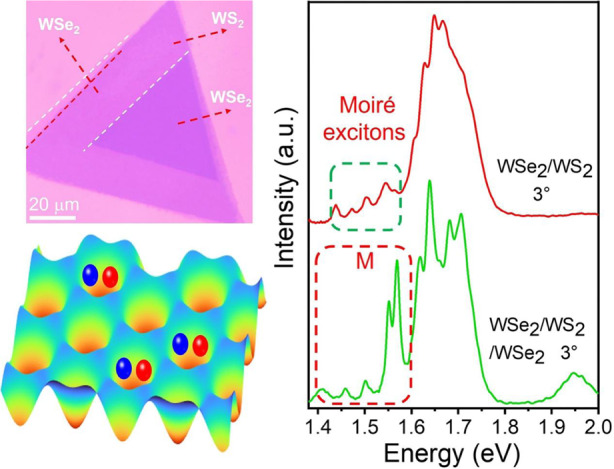

## Introduction

Recently, the twisted van der Waals heterostructured superlattices have attracted significant attention as they provide a powerful and attractive platform for exploring the new physics of novel condensed matter^1–6^. Vertically stacked 2D materials can generate periodic moiré superlattices due to lattice mismatches or twist angles^7^. The moiré potential in the moiré superlattice dominates the kinetic energy within the mini-Brillouin zone, which changes the electronic band structure in the heterojunction^8–10^, and induces strongly correlated quantum phenomena: including strongly correlated insulators^11–14^, superconductivity^15^, moiré excitons^16–18^, moiré phonons^19,20^, magnetism^21^. Moiré superlattices in twisted 2D material heterojunctions offer opportunities for the development of many-body physics^22,23^, which will help to drive the development of novel quantum devices^24^. The periodic moiré potentials induced by moiré superlattices in van der Waals heterojunctions can trap interlayer excitons to generate moiré exciton arrays^25–27^. The tunability of the moiré potential opens a new avenue for quantum manipulation of quasiparticles in quantum optics. Recently, the moiré excitons have been reported in a twisted MoSe_2_/WSe_2_ heterojunction, and multiple interlayer exciton resonance phenomena have been observed. They attribute these resonances to the state of exciton on the ground and the state of excitation related to moiré potential^28^. Such moiré superlattices can be applied to quantum emitter arrays^29^. However, the relationship between the modulation effect of moiré superlattices on the properties of moiré excitons and the number of twisted layers has yet to be further studied, particularly for 2D twisted angle heterojunctions with more than two layers.

In this work, we utilize the layer degrees of freedom to investigate the localization of moiré excitons. We report the observation of multiple exciton resonances in a high-quality hexagonal boron nitride (hBN)-encapsulated in the twisted heterotrilayer. The WSe_2_/WS_2_/WSe_2_ heterotrilayer has two type-II band alignments that form two overlapping moiré potentials at the twisted WSe_2_/WS_2_ interface. The synergy of the two moiré potentials enables the moiré excitons at the interface very localized, manifesting in the form of multiple sharp emission lines, in sharp contrast to the moiré excitonic behavior of the twisted WSe_2_/WS_2_ heterobilayer. Additionally, comparing the variation of the laser power and temperature of the twisted-angle heterojunction with different layers, further proved that the formation of double moiré fringes at the WSe_2_/WS_2_/WSe_2_ heterotrilayer interface will induce a deeper and narrower moiré potential to localize excitation. Simultaneously, the magneto-optical spectroscopy results show that the distinguishable *g*-factor is a result of the exciton confinement in the potential created by the moiré pattern. Our results offer a new way to regulate the localization of moiré excitons in twisted-angle heterostructures, promising single-photon emission of excitons to advance the application of moiré superlattices in quantum devices.

## Results

### Schematic and WSe_2_/WS_2_/WSe_2_ heterotrilayer

**Fig. 1:**
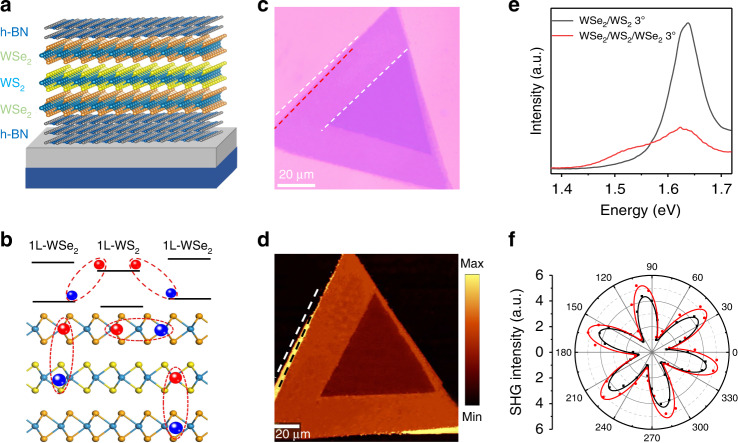
Moiré superlattice and interlayer exciton emission in a WSe_2_/WS_2_/WSe_2_ heterotrilayer. **a** Schematic illustration of a vertically stacked WSe_2_/WS_2_/WSe_2_ twisted-angle heterotrilayer with hexagonal boron nitride (h-BN) on a SiO_2_/Si substrate. The twisted WSe_2_/WS_2_/WSe_2_ heterotrilayer is a system composed of two-layer WSe_2_ and one-layer WS_2_. The top and bottom sheets are aligned, and the middle sheet is rotated by a *θ* angle of approximately 3° relative to the other two sheets. **b** Schematic diagram of the alignment of type II bands in the WSe_2_/WS_2_/WSe_2_ heterotrilayer. The energy levels are represented by black solid lines, and the interlayer excitons and intralayer excitons are marked by dashed ellipses. **c** Optical microscopy image of the WSe_2_/WS_2_/WSe_2_ heterotrilayer, with the heterojunctions encapsulated with flakes of BN (Fig. S1). **d** Raman mapping of a twisted WSe_2_/WS_2_/WSe_2_ heterotrilayer. **e** The PL spectra of heterojunctions with different layers at room temperature. **f** Measured and fitted the SHG signals of the top WSe_2_ monolayer and middle WS_2_ monolayer regions of the sample, which confirm the 3° twist angle between the top and middle layers

In the twisted heterostructures of 2D materials, the periodic moiré superlattices can be formed by tuning the lattice mismatch and the interlayer twist angle (*θ*). The periodicity of the moiré superlattice changes correspondingly with the twist angles, and its electronic structure and energy band structure also changes, resulting in multiple planar exciton miniature energy bands. Moiré exciton bands provide a novel platform for exploring and controlling excited states of matter. Figure 1a shows the schematic of the h-BN-encapsulated WSe_2_/WS_2_/WSe_2_ heterotrilayer, which includes three different regions in the same device: 1L-WSe_2_, 1L-WSe_2_/WS_2_ and WSe_2_/WS_2_/WSe_2_ heterotrilayer. The WSe_2_/WS_2_/WSe_2_ heterotrilayer have two type II band alignment, which results in the formation of spatially indirect interlayer excitons, with electrons and holes that reside in the WS_2_ and WSe_2_ layers, respectively (Fig. 1b). At the same time, the twisted WSe_2_/WS_2_/WSe_2_ heterotrilayer is a system composed of two-layer WSe_2_ and one-layer WS_2_. The top and bottom sheets are aligned, and the middle sheet is rotated by a *θ* angle of approximately 3° relative to the other two sheets. Interlayer torsion angles create periodic moiré superlattices at the interface that trap and spatially confine excitons. In the twisted WSe_2_/WS_2_/WSe_2_ heterotrilayer, the torsion angle of the three layers will generate two periodic moiré fringes and then form super moiré fringes, leading to new quantum phenomena.

Figure 1c shows the optical image of the WSe_2_/WS_2_/WSe_2_ heterotrilayer, we can distinguish monolayer, bilayer twist areas and trilayer twist areas. The vdW heterostructures were prepared via polymethyl methacrylate (PMMA)-assisted transfer method. Our heterobilayer samples encapsulated by hexagonal boron nitride (hBN) (Fig. S1). The Raman mapping (Fig. 1d) was used to confirm the quality of the twisted WSe_2_/WS_2_/WSe_2_ heterotrilayer. The uniformity of Raman mapping signal intensity can confirm spatial homogeneity over the micrometer length scale, which is mainly attributed to our dry transfer method and annealing treatment. At the same time, the Raman spectra of the heterostructures with different layers further proved that the twisted WSe_2_/WS_2_/WSe_2_ heterotrilayer was successfully prepared (Fig. S2). As shown in Fig. 1e, the PL spectra of WSe_2_/WS_2_/WSe_2_ heterotrilayer with different layers at room temperature, and it can be found that the PL peak has a red shift and that its intensity decreases with the increase of the number of layers. Also, the PL spectra display a new emission peak at ~1.52 eV in the WSe_2_/WS_2_/WSe_2_ heterotrilayer with twist angles of 3°, which is attributed to the emission from the interlayer excitons. The relative twist angle between the top and middle sheets of the sample was determined optically using polarization-dependent second-harmonic-generation measurements^30^. Figure 1f shows the polarization-dependent PL of the top and a middle sheet of WSe_2_ and WS_2_, from which can determine a rotation of the principal axis of 3° ± 0.2° between the layers.

### Moiré exciton localization in a WSe_2_/WS_2_/WSe_2_ Heterotrilayer

**Fig. 2:**
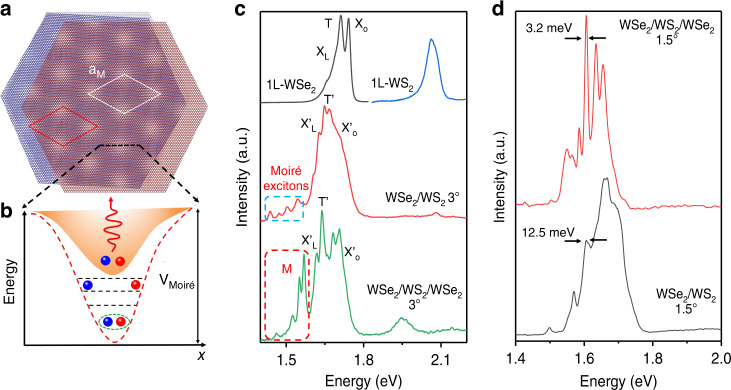
Moiré exciton localization in a twisted heterotrilayer. **a** Schematics of moiré superlattices formed in the twisted WSe_2_/WS_2_/WSe_2_ heterotrilayer. **b** The moiré superlattice leads to a periodic moiré potential in the twisted WSe_2_/WS_2_/WSe_2_ heterotrilayer, which can trap excitons in the moiré traps. The deeper the moiré potential, the more excitons can be trapped. **c** Representative PL spectra of 1L-WSe_2_, 1L-WS_2_, WSe_2_/WS_2_ heterobilayer with a twist angle of 3° and the WSe_2_/WS_2_/WSe_2_ heterotrilayer with a twist angle of 3°. The PL intensity of the heterostructure is multiplied by a factor of three to facilitate comparison with the PL spectrum of 1L-WSe_2_. The T’ and X_O_ peaks are assigned to emissions from the trion and exciton of the 1L-WSe_2_, respectively. The low-energy additional peaks representing moiré excitons are indicated by the red dotted box (M). **d** Representative PL spectra of WSe_2_/WS_2_ heterobilayer with a twist angle of 1.5° and the WSe_2_/WS_2_/WSe_2_ heterotrilayer with a twist angle of 1.5°. Line widths of moiré excitons of 12.5 meV and 3.2 meV are obtained for WSe_2_/WS_2_ heterobilayer and the WSe_2_/WS_2_/WSe_2_ heterotrilayer

In twisted heterostructures of 2D materials, by adjusting the lattice mismatch and the interlayer twist angle (*θ*), a moiré superlattice can be formed, resulting in a periodic moiré potential to trap excitons. The WSe_2_/WS_2_/WSe_2_ heterotrilayer has two type-II band alignments, with the conduction band minimum located in the WS_2_ layer and the valance band maximum in the top and bottom WSe_2_ layer. When the top and bottom WSe_2_ layers are slightly misaligned with the middle WS_2_ layer, two interfering moiré patterns are formed at the WSe_2_/WS_2_ interface (Fig. 2a). The moiré superlattice leads to band folding in the mini-Brillouin region and creates moiré exciton bands that capture more moiré excitons^31^. The optical spectra of the moiré exciton change systematically in a way that suggests the moiré coupling is highly interfacial, strongly confined at the WSe_2_/WS_2_ interface and barely affects the next neighboring WSe_2_ layer(s). The added WSe_2_ layer(s) could modify moiré excitons in the WSe_2_ layer interfacing WS_2_, resulting in a significant increase in the resonance energy separations between moiré excitons (Fig. 2b). Therefore, we believe that changing the number of stacking layers can regulate the interface moiré exciton.

To investigate the localized effects of the layer degree of freedom on moiré excitons, we performed micro-photoluminescence measurements on the twisted WSe_2_/WS_2_ heterobilayer and the WSe_2_/WS_2_/WSe_2_ heterotrilayer at 6 K under a low excitation power. The PL spectrum of 1L-WSe_2_ is well known with two well-separated and narrow excitonic emissions, which can be attributed to neutral free excitons (X_o_) at 1.742 eV and free charged excitons (T), at 1.711 eV, which is consistent with previously reported results^32^ (Fig. 2c). By contrast, the PL spectrum of the twisted WSe_2_/WS_2_ heterobilayer is strikingly different from that of 1L-WSe_2_. Figure 2c shows the PL spectrum of the WSe_2_/WS_2_ heterobilayer with a twist angle of 3°, which can find that the PL spectrum of WSe_2_/WS_2_ heterostructure has additional fine peaks (M) on the lower-energy side beside the neutral free excitons peak (X_o_’) at 1.651 eV and the trion peak (T’) at 1.708 eV. This suggests the existence of a periodic moiré superlattice in the twisted WSe_2_/WS_2_ heterobilayer, creating moiré traps at the interface that traps the excitons in them, modulating their energy levels and causing them to split. To further examine the influence of the moiré potential on interlayer excitons, we prepared the WSe_2_/WS_2_ heterobilayer with different twist angles. By adjusting the twist angle, the moiré superlattice period can be tuned. With the increase of the twist angle, the moiré superlattice period decreases, resulting in an increase in the moiré potential, which modulates more exciton energies and further form moiré excitons. Figure S5 shows the PL spectra of the WSe_2_/WS_2_ heterobilayer with twist angles of 3 and 1.5°, respectively. We focus on the WSe_2_/WS_2_ heterobilayer with a twist angle of 3°. The central emission energies extracted are 1.545 eV, 1.503 eV, 1.472 eV, 1.437 eV, respectively. Compared to the WSe_2_/WS_2_ heterobilayer with twist angles of 1.5°, the splitting peaks are shifted towards lower energies. This is mainly because with the twist angle increases, the depth of moiré potential increases, capturing more excitons to form the splitting peaks. Our experimental results further proved that the stack of the WSe_2_/WS_2_ heterobilayer with different twist angles can effectively improve the moiré potential. Meanwhile, the change of these exciton peaks with the twist angle further proves that excitonic states with low-energy emissions originate from moiré excitons.

The construction of a twisted-angle WSe_2_/WS_2_/WSe_2_ heterotrilayer can form double moiré fringes, which enable highly localized moiré excitons. We performed micro-photoluminescence measurements on the WSe_2_/WS_2_/WSe_2_ heterotrilayer with a twist angle of 3° at 6 K under a low excitation power (Fig. 2c). Compared to the WSe_2_/WS_2_ heterobilayer with a twist angle of 3°, the intensity of the intralayer excitons and moiré exciton peaks in the twisted-angle WSe_2_/WS_2_/WSe_2_ heterotrilayer are increased by 3–5 times. The localization of moiré excitons in a supermoiré-induced potential trap gives rise to a sharp emission peak. To further verify the localized effects of twist angle on moiré excitons, we prepared the WSe_2_/WS_2_/WSe_2_ heterotrilayer with different twist angles. Figure 2d shows the PL spectra of the WSe_2_/WS_2_ heterobilayer with twist angles of 1.5°, which can also find the same moiré excitons localization phenomeno. The considerably narrow line width of the localized moiré exciton peaks (average line width = 3.2 meV, Fig. 2d, top) compared to that of the moiré exciton peaks without localization (average line width = 12.5 meV, Fig. 2d, bottom) (Fig. S6). The localization of moiré excitons is mainly due to the double moiré fringes formed at the interface of the WSe_2_/WS_2_/WSe_2_ heterotrilayer, resulting in deeper and narrower moiré potential traps. In a highly confined moiré potential well can lead to an increase in the auger recombination rate and an enhancement of exciton-exciton interactions, leading to the localization of excitons^33,34^.

**Fig. 3:**
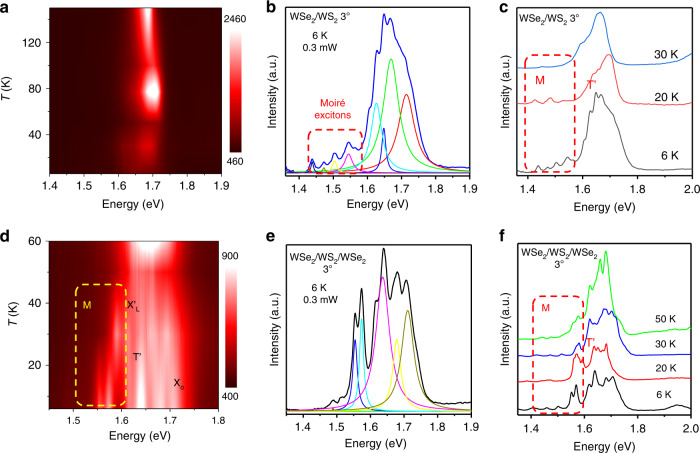
Temperature dependence of moiré excitons in the twisted WSe_2_/WS2/WSe_2_ heterotrilayer. **a**, **b** Contour plot of the temperature-dependent PL in the WSe_2_/WS_2_ heterobilayer with a twist angle of 3°. PL spectrum of WSe_2_/WS_2_ heterobilayer at 6 K under an excitation power density of 0.3 mW extracted from the contour plot. **c** The normalized PL spectra of the twisted WSe_2_/WS_2_ heterobilayer with a twist angle of 3° at various temperatures ranging from 6 to 30 K. **d**, **e** Contour plot of the temperature-dependent PL in the WSe_2_/WS_2_/WSe_2_ heterotrilayer with a twist angle of 3°. The PL spectrum shows a splitting phenomenon at 6 K under an excitation power density of 0.3 mW extracted from the contour plot. **f** The normalized PL spectra of the twisted WSe_2_/WS_2_/WSe_2_ heterotrilayer with twist angle of 3° at various temperatures ranging from 6 to 50 K

Temperature dependence of the integrated PL intensity provides key insight into the localized nature of moiré excitons. To further demonstrate that we observed moiré excitons in the twisted WSe_2_/WS_2_/WSe_2_ heterotrilayer, we studied the PL intensity as a function of temperature. The contour map of the temperature dependence of the PL spectra shown in Fig. 3a displays the origins of additional spectral fine structures in the WSe_2_/WS_2_ heterobilayer with a twist angle of 3°. Figure 3b shows the PL spectrum of the twisted WSe_2_/WS_2_ heterobilayer at 6 K and fitted with gaussian functions. It can be clearly found that multiple splitting peaks at 1.4 and 1.57 eV are different from the monolayer WSe_2_ exciton peaks, which is mainly caused by the moiré potential trapping excitons in the twisted WSe_2_/WS_2_ heterobilayer. Figure 3c presents the PL spectra from 6 to 30 K obtained from the horizontal line cut of the contour map. The red-shift of the PL peaks with increasing temperature are owed to the temperature-dependent bandgap shift.

At the same time, Fig. 3c shows the temperature dependence of the PL intensities of the moiré excitons and the trion (T’) state. With the temperature increases, the PL intensity of the moiré excitons states rapidly decreases and disappears at temperatures higher than ∼30 K. This is mainly because the thermal energy is greater than the trapping potential energy, and the exciton-bound state undergoes thermal dissociation assisted by thermal excitation. The experimental results can be explained by the temperature dependence of the integrated PL intensity assisted by the thermal excitation^35,36^:

$$I\left(T\right)=I\left(0\right)\frac{1}{1+A\,{exp} {\left(-\frac{E}{{k}_{B}T}\right)}^{{\prime} }}$$

*I* (0) is the PL intensity at the lowest temperature limit, *k*_B_ is the Boltzmann constant, *A* refers to the parameter, *E* is the activation energy corresponding to the depth of the moiré potential, and *T* is the temperature. The relationship between the PL intensity and temperature can be seen through this formula, and it can be found that the moiré potential has an influence on the thermal dissociation and thermal excitation of the exciton bound state. The moiré-trapped state has moiré potential confinement energy than the trion (T’) state, so T’ can be delocalized more easily with a thermally assisted process. As shown in Fig. 3c, the intensity of the trion (T’) PL decreases more rapidly and can no longer decompose when the temperature is greater than 20 K. The T’ state exhibits a faster radiative recombination process. As a result of the confinement of the moiré potential, the PL intensity of the moiré excitons decreases relatively slowly. These findings are consistent with the previously reported results of the moiré excitons, implying that the extra peaks come from the moiré potential.

The contour map of the temperature dependence of the PL spectra shown in Fig. 3d reveals the origins of additional spectral fine structures in the WSe_2_/WS_2_/WSe_2_ heterotrilayer with a twist angle of 3°. Figure 3e shows the PL spectrum of the twisted WSe_2_/WS_2_/WSe_2_ heterotrilayer at 6 K and fitted with gaussian functions, which can be found that multiple splitting peaks at 1.4 and 1.6 eV are different from the WSe_2_/WS_2_ heterobilayer exciton peaks. The intensity of the intralayer excitons and moiré exciton peaks in the twisted-angle WSe_2_/WS_2_/WSe_2_ heterotrilayer are increased by 3–5 times. The localization of moiré excitons in a supermoiré-induced potential trap gives rise to a sharp emission peak. This is mainly due to the formation of double moiré fringes at the WSe_2_/WS_2_/WSe_2_ heterotrilayer, which results in deeper and narrower moiré potential traps, leading to the localization of moiré excitons. To further prove that the localized moiré exciton states have a deeper moiré potential, we extracted the exciton peaks as a function of temperature. Figure 3f presents the PL spectra from 6 to 50 K obtained from the horizontal line cut of the contour map. The intensity of the trion (T’) PL decreases more rapidly and can no longer decompose when the temperature is greater than 30 K. Compared to the WSe_2_/WS_2_ heterobilayer with a twist angle of 3°, the PL intensity of the moiré excitons state of the WSe_2_/WS_2_/WSe_2_ heterotrilayer decreases slowly and disappears at temperatures higher than ∼50 K. Our experimental results further demonstrate that localized moiré excitons have a deeper trapping potential and require more thermal energy to delocalize the excitons.

## Discussion

**Fig. 4:**
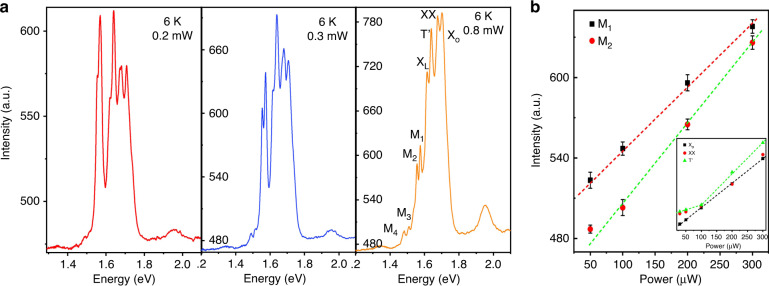
Power-dependence of moiré excitons in twisted WSe_2_/WS_2_/WSe_2_ heterotrilayer. **a** PL spectra of the twisted WSe_2_/WS_2_/WSe_2_ heterotrilayer as a function of excitation power under 532 nm laser excitation at 6 K. **b** The PL intensity of M_1_, M_2_ peaks as a function of excitation power under 532 nm laser excitation at 6 K. The inset shows the PL intensity of the T^’^, XX and X_o_ peaks as a function of excitation power

Another important effect of the moiré potential is the excitation power dependence of the PL spectra, we investigated the power-dependent PL spectrum in the twisted WSe_2_/WS_2_/WSe_2_ heterotrilayer under 532 nm laser excitation at 6 K. Figure 4a shows the PL spectra of the WSe_2_/WS_2_/WSe_2_ heterotrilayer at different power densities at 6 K. At low excitation intensities below ∼0.3 mW, we can observe that the PL spectrum shows that the moiré excitonic peaks (M_1_, M_2_, M_3_, and M_4_) dominate the spectrum. With the excitation power increases, the moiré exciton peaks at lower energy levels gradually disappear and the peak widths become larger. At the same time, we also found that with the increase of excitation power (more than 1 mW), the moiré exciton peaks changed from multiple small splitting peaks to the main peak dominated by intralayer excitons, and the intensity of high energy level (X’_o_ and T’) peaks increased. The results indicate that at low power (less than 0.3 mW), the splitting peaks of the PL spectrum are mainly due to the capture of excitons by the moiré potential. With the increase of power, exciton filling goes from low energy level to high energy level sequentially. The moiré flat bands are filled and gradually reach saturation, losing its modulation effect on excitons.

To further observe the evolution trend of moiré excitons with excitation power, we extracted the PL intensities of moiré exciton peaks and intralayer exciton peaks as a function of power (Fig. 4b). The shapes with different colors indicate different peak positions extracted. We can find that the moiré excitons increase linearly with power at the low excitation power and quickly saturate. With the increase of power, excitons are sequentially filled from low energy level to high energy level, the moiré exciton peaks gradually disappear, and the energy is transferred to the intralayer exciton peaks of high energy level. Meanwhile, we find that the interlayer exciton peaks (M_1_, M_2_, M_3_, and M_4_) blue-shift with the increase of power, which is mainly caused by repulsive dipole-dipole interactions. However, intralayer excitons do not move with the increase of power (Fig. S9). To further distinguish intralayer excitons, we extracted the PL intensities of intralayer exciton peaks as a function of power. With the increase of power, we find that the intensity of the neutral exciton (X_o_) peak increases linearly with power, whereas the intensity of charged exciton peak (T’) increases nonlinearly with power, which is consistent with the previous reports^37^.

In a twisted-angle heterojunction, an ideal moiré superlattice has C_3_ symmetry and it emits the same amount of co-polarized and cross-polarized light. When linearly polarized light is used to excite an ideal moiré superlattice, the direction of the linearly polarized light has no effect on the emitted light^38,39^. Therefore, the linear polarization can be used to demonstrate how close the WSe_2_/WS_2_/WSe_2_ heterotrilayer is to being an ideal moiré superlattice. Figure S11a, b shows the linearly polarized spectrum of the monolayer WSe_2_, which can be found that the X_o_ exciton of the monolayer WSe_2_ do not change significantly with the change of the linear polarization direction *ϕ* (Supplementary Fig. S10a). This is mainly because the monolayer WSe_2_ has C_3_ symmetry and its photoluminescence does not exhibit linear polarization.

Figure S11c, d shows the linear polarization dependence of the twisted, which can be found that the excitons of the twisted WSe_2_/WS_2_/WSe_2_ heterotrilayer are slightly affected by the linear polarization (Supplementary Fig. S10b). In fact, the highest degree of linear polarization in the WSe_2_/WS_2_/WSe_2_ heterotrilayer (Fig. S11d) was around (10 ± 8)% at the emission energy of 1.689 eV. The excitons of the twisted WSe_2_/WS_2_/WSe_2_ heterotrilayer are affected by the linear polarization, which is mainly because the strain and relaxation in the heterojunction lead to the uneven distribution of the positions of the emitted co-polarized light and cross-polarized light. Moreover, the degree of linear polarization in our twisted heterojunction is much lower than that of strained heterostructures reported in the literature, indicating that the C_3_ symmetry is preserved in our sample^40^. Therefore, the shape of the moiré superlattice formed in the twisted heterojunction is fundamentally regular. In addition, an important effect of the moiré pattern is the imposition of spatially varying optical selection rules^38^. To verify that we are observing a moiré exciton phenomenon, we provide evidence for the existence of moiré superlattices in the twisted WSe_2_/WS_2_/WSe_2_ heterotrilayer using the alternating circularly polarized photoluminescence. Figure S11e, f shows PL spectra of the σ^+^σ^+^ and σ^+^σ^−^ configurations, which can be clearly find that there is a clear cross-polarization at 1.4–1.6 eV. The generation of this cross-polarization is mainly due to the spatial variation caused by atomic rotational symmetry, and the relative positions of atoms in different positions in real space are different, thus affecting the optical selection rule^41^. This cross-polarization phenomenon provides further evidence for the existence of a moiré superlattice in our twisted-angle heterotrilayer. We also carried out density functional theory studies to confirm the existence of moiré potentials in twisted-angle heterotrilayer (Fig. S13). The highest valence band width is only 1 meV, indicating a flat valence band behavior in the twisted WSe_2_/WS_2_/WSe_2_ moiré superlattice. These calculated results give a good understanding of the splitting peak spacing of moiré excitons observed in our experiments.

**Fig. 5:**
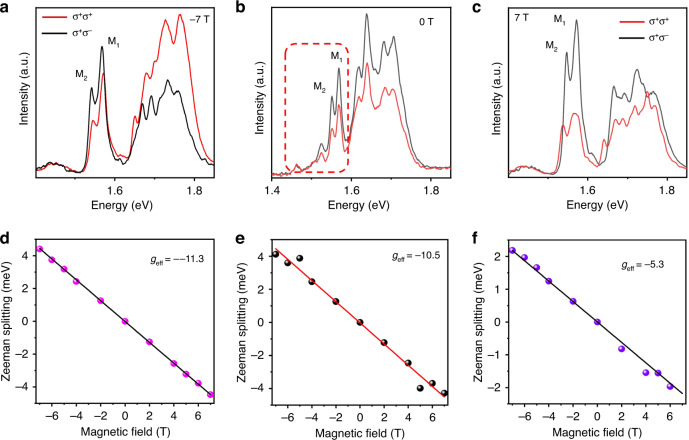
Magneto-PL spectroscopy of moiré excitons. **a**–**c** Circularly polarized photoluminescence (PL) spectra of the twisted WSe_2_/WS_2_/WSe_2_ heterotrilayer at −7, 0 and 7 T. **d**–**f** Zeeman splitting of the circularly polarized photoluminescence (*ΔE* = *E*_*σ+*_ *− E*_*σ−*_) as a function of the magnetic field. The corresponding effective *g*-factors for the moiré excitons and neutral exciton are −11.3 ± 0.5, −10.5 ± 0.2 and −5.3 ± 0.5, respectively

To further support a role of the moiré potential in producing these effects, we performed magneto-photoluminescence spectroscopy to determine the Landé *g*-factor of trapped interlayer excitons. We can define the Zeeman splitting between the PL peaks as *ΔE* = *E*_*σ+*_ − *E*_*σ−*_, which is to be distinguished from the valley Zeeman splitting. Figure 5a–c shows the circularly polarized PL spectra at various magnetic fields (−7, 0, and 7 T) at 10 K. The PL spectra are resolved with the σ^+^ and σ^−^ components, which correspond to the signals from the K^+^ and K^−^ valleys, respectively. The σ^+^ and σ^−^ components of the X_o_ (neutral exciton) peak are not offset without a magnetic field in the twisted WSe_2_/WS_2_/WS_2_ heterotrilayer, whereas the moiré excitons show a slight difference between the PL intensities of the σ^+^ and σ^−^ components. With increasing magnetic field, the degree of valley polarizability of moiré excitons greatly increases, while that of X_o_ peak increases slightly.

Figure 5d–f shows the magnetic field-dependent Zeeman splitting of the moiré excitons and X_o_ peaks, where the Zeeman splitting is defined as the peak energy difference between the σ^+^ and σ^−^ components. The Zeeman splittings of both the X_o_ and moiré excitons peaks depend linearly on the magnetic field (from −7 to 7 T). The slope of the Zeeman splitting for the X_o_ peak was estimated to be −5.3 ± 0.5 meV/T. It should be noted that that the slope of the Zeeman splitting for the moiré excitons peaks (−11.3 ± 0.5, −10.5 ± 0.3 meV/T) are different from that obtained for the X_o_ peak. We can find that the *g*-factor of moiré excitons is basically the same and very different from the *g*-factor of the X_o_ peak. The *g*-factor of an interlayer exciton is therefore representative of its valley configuration and its valley magnetic moment. Although inhomogeneity of moiré traps gives rise to a distribution of charged exciton and moiré exciton peak energies, the peak energy shifts as a function of magnetic field are nearly the same for all moiré exciton. This behavior is characteristic of excitons trapped in a moiré potential. Therefore, it can be shown by the *g*-factor that these excitons are trapped by the moiré potential rather than defects.

In conclusion, we have presented a novel moiré superlattice system of twisted-angle 2D heterojunctions. By utilizing the layer degrees of freedom, we created a twisted WSe_2_/WS_2_/WSe_2_ heterotrilayer that generates two periodic moiré fringes. The combination of these two moiré potentials results in highly localized moiré excitons, shown as multiple sharp emission lines. The rotation angle can be adjusted to tune the local moiré excitons and the effect of the moiré potential on excitons is further demonstrated through changes in laser power and temperature. This demonstrates that localization-enhanced moiré superlattices in twisted van der Waals heterojunctions can be fabricated and provide a fascinating platform for exploring new quantum phenomena.

## Materials and methods

### Fabrication of moiré heterostructures

The monolayer WSe_2_ (WS_2_) films were synthesized by a typical CVD growth method. The WO_3_ (20 mg)) was selected as the solid source for the one-step growth. 50 mg of Se (S) powder was placed upstream of the tube furnace. Before heating, the system was cleaned with a high-purity Ar gas and maintained for about 30 min. Then, the furnace was heated to 830 °C and kept at this temperature for 20 min. The S powder was put in the upstream region with a temperature of 190 °C. H_2_/Ar mixture flow was used as carrier gas. After the growth, the furnace was cooled down to room temperature naturally.

The twisted WSe_2_/WS_2_/WSe_2_ heterotrilayer were fabricated by a wet-transfer technique with a polymethyl methacrylate (PMMA) film. One layer was transferred onto the other. The top WSe_2_ monolayer was then stacked onto the bottom monolayer with the crystal axes rotationally aligned under an optical microscope. Finally, the SHG was used to determine the rotation angle between the two monolayers, and the excitation light source of the SHG signal is a 1064 nm pulsed laser. The samples were annealed in a high vacuum at 300 °C for 3 h.

### Optical measurements

For steady-state photoluminescence measurements, the sample was performed on the WITec Alpha 300 R system and excited using a continuous-wave 532-nm laser focused to a spot size of 1.5 μm. The sample temperature was kept at 6 K. The pressure of the low-temperature test system is below 10^–5^ pa, and the temperature is cooled by compressing helium gas. When the temperature is stable at 6 K, the PL spectrum test of the sample is carried out. The model of the cryogenic refrigeration system is C04-005-044, which comes from the Cryo Industries of America.

## Supplementary information

Supplementary information

## References

[1] Cao Y (2018). Correlated insulator behaviour at half-filling in magic-angle graphene superlattices. Nature.

[2] Yang J (2015). Optical tuning of exciton and trion emissions in monolayer phosphorene. Light Sci. Appl..

[3] Zheng H (2023). Evidence for interlayer coupling and moiré excitons in twisted WS_2_/WS_2_ homostructure superlattices. Nano Res..

[4] Naik MH (2022). Intralayer charge-transfer moiré excitons in van der Waals superlattices. Nature.

[5] Wang HF (2022). Intrinsic superflat bands in general twisted bilayer systems. Light Sci. Appl..

[6] Ye T (2022). Nonvolatile electrical switching of optical and valleytronic properties of interlayer excitons. Light Sci. Appl..

[7] Li S (2022). Dynamic control of moiré potential in twisted WS_2_-WSe_2_ heterostructures. Nano Res..

[8] Sharpe AL (2019). Emergent ferromagnetism near three-quarters filling in twisted bilayer graphene. Science.

[9] Chen GR (2020). Tunable correlated chern insulator and ferromagnetism in a moiré superlattice. Nature.

[10] White SJU (2022). Electrical control of quantum emitters in a van der Waals heterostructure. Light Sci. Appl..

[11] Polshyn H (2020). Electrical switching of magnetic order in an orbital chern insulator. Nature.

[12] Regan EC (2020). Mott and generalized Wigner crystal states in WSe_2_/WS_2_ moiré superlattices. Nature.

[13] Xian LD (2019). Multiflat bands and strong correlations in twisted bilayer boron nitride: doping-induced correlated insulator and superconductor. Nano Lett..

[14] Tang YH (2020). Simulation of Hubbard model physics in WSe_2_/WS_2_ moiré superlattices. Nature.

[15] Cao Y (2018). Unconventional superconductivity in magic-angle graphene superlattices. Nature.

[16] Chen DX (2022). Tuning moiré excitons and correlated electronic states through layer degree of freedom. Nat. Commun..

[17] Wu B (2022). Evidence for moiré intralayer excitons in twisted WSe_2_/WSe_2_ homobilayer superlattices. Light Sci. Appl..

[18] Huang D (2022). Excitons in semiconductor moiré superlattices. Nat. Nanotechnol..

[19] Quan JM (2021). Phonon renormalization in reconstructed MoS_2_ moiré superlattices. Nat. Mater..

[20] Kim J (2022). Anomalous optical excitations from arrays of whirlpooled lattice distortions in moiré superlattices. Nat. Mater..

[21] Qiu ZZ (2021). Visualizing atomic structure and magnetism of 2D magnetic insulators via tunneling through graphene. Nat. Commun..

[22] Tan, Q. et al. Layer-dependent correlated phases in WSe_2_/MoS_2_ moiré superlattice. *Nat. Mater*. 10.1038/s41563-023-01521-4 (2023). 10.1038/s41563-023-01521-4 37069294

[23] Miao S (2021). Strong interaction between interlayer excitons and correlated electrons in WSe_2_/WS_2_ moiré superlattice. Nat. Commun..

[24] Lamas-Linares A, Howell JC, Bouwmeester D (2001). Stimulated emission of polarization-entangled photons. Nature.

[25] Karni O (2022). Structure of the moiré exciton captured by imaging its electron and hole. Nature.

[26] Turunen M (2022). Quantum photonics with layered 2D materials. Nat. Rev. Phys..

[27] Zhang L (2021). Van der Waals heterostructure polaritons with moiré-induced nonlinearity. Nature.

[28] Seyler KL (2019). Signatures of moiré-trapped valley excitons in MoSe_2_/WSe_2_ heterobilayers. Nature.

[29] Sergeev AA (2020). Tailoring spontaneous infrared emission of HgTe quantum dots with laser-printed plasmonic arrays. Light Sci. Appl..

[30] Zhao SL (2021). Anisotropic moiré optical transitions in twisted monolayer/bilayer phosphorene heterostructures. Nat. Commun..

[31] Anđelković M (2020). Double Moiré with a twist: supermoiré in encapsulated graphene. Nano Lett..

[32] Ye ZL (2018). Efficient generation of neutral and charged biexcitons in encapsulated WSe_2_ monolayers. Nat. Commun..

[33] Pietryga JM (2008). Evidence for barrierless Auger recombination in PbSe nanocrystals: A pressure-dependent study of transient optical absorption. Phys. Rev. Lett..

[34] Robel I (2009). Universal size-dependent trend in Auger recombination in direct-gap and indirect-gap semiconductor nanocrystals. Phys. Rev. Lett..

[35] Shibata H (1998). Negative thermal quenching curves in photoluminescence of solids. Jpn. J. Appl. Phys..

[36] Fang YT (2015). Investigation of temperature-dependent photoluminescence in multi-quantum wells. Sci. Rep..

[37] Paur M (2019). Electroluminescence from multi-particle exciton complexes in transition metal dichalcogenide semiconductors. Nat. Commun..

[38] Yu HY (2017). Moiré excitons: from programmable quantum emitter arrays to spin-orbit-coupled artificial lattices. Sci. Adv..

[39] Wu FC, Lovorn T, MacDonald AH (2018). Theory of optical absorption by interlayer excitons in transition metal dichalcogenide heterobilayers. Phys. Rev. B.

[40] Bai YS (2020). Excitons in strain-induced one-dimensional moiré potentials at transition metal dichalcogenide heterojunctions. Nat. Mater..

[41] Tran K (2019). Evidence for moiré excitons in van der Waals heterostructures. Nature.

