# Supplementary material for: Localization-enhanced moiré exciton in twisted transition metal dichalcogenide heterotrilayer superlattices
Source: Light Sci Appl. 2023 May 12;12:117. doi: 10.1038/s41377-023-01171-w (PMC10182042; doi:10.1038/s41377-023-01171-w)
Supplement: Supplementary file 1 — Supplementary information [file 41377_2023_1171_MOESM1_ESM.pdf]

Supplementary information for

Localization-enhanced moiré exciton in twisted transition metal dichalcogenide heterotrilayer superlattices

Haihong Zheng[1,2,], Biao Wu[1,2,], Shaofei Li[1], Junnan Ding[1], Jun He[1], Zongwen Liu[3,4], Chang-Tian Wang[5,6,7], Jian-Tao Wang[5,6,7], Anlian Pan[8,*] & Yanping Liu[1,2,9*]

1. *School of Physics and Electronics, Hunan Key Laboratory for Super-microstructure and Ultrafast Process, Central South University, 932 South Lushan Road, Changsha, Hunan 410083, China*
2. *State Key Laboratory of High-Performance Complex Manufacturing, Central South University, 932 South Lushan Road, Changsha, Hunan 410083, China*
3. *School of Chemical and Biomolecular Engineering, The University of Sydney, NSW 2006, Australia*
4. *The University of Sydney Nano Institute, The University of Sydney, NSW 2006 Australia*
5. *Beijing National Laboratory for Condensed Matter Physics, Institute of Physics, Chinese Academy of Sciences, Beijing 100190, China*
6. *School of Physical Sciences, University of Chinese Academy of Sciences, Beijing 100049, China*
7. *Songshan Lake Materials Laboratory, Dongguan, Guangdong 523808, China*
8. *Hunan Institute of Optoelectronic Integration, College of Materials Science and Engineering, Hunan University, Changsha, Hunan 410082, China*
9. *Shenzhen Research Institute of Central South University, Shenzhen 518000, China*

* Correspondence and requests for materials should be addressed to email:
liuyanping@csu.edu.cn; anlian.pan@hnu.edu.cn

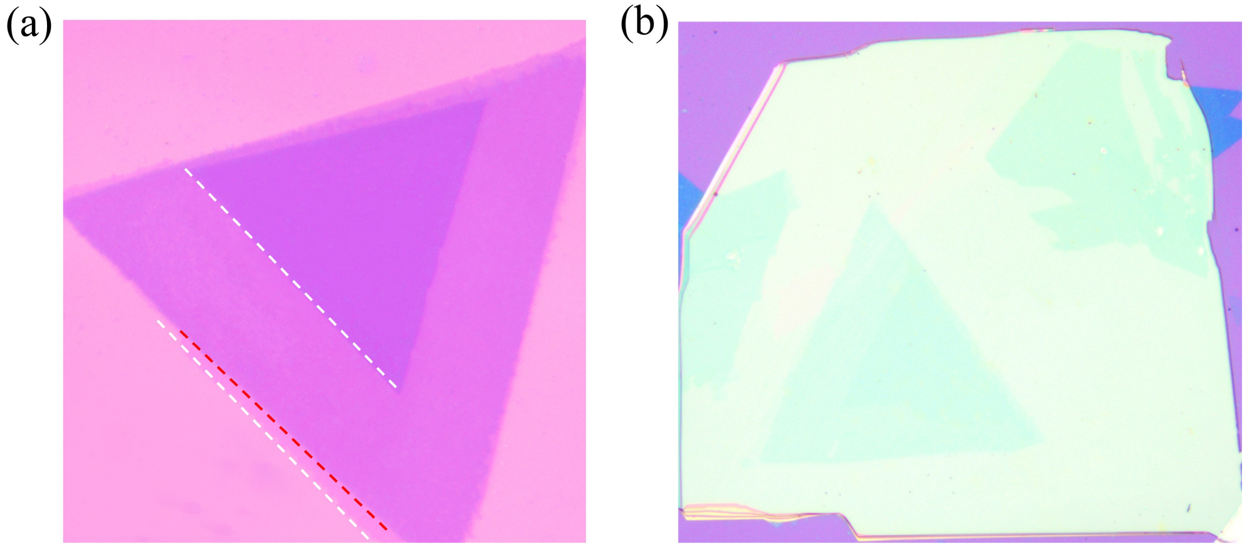

Figure S1 (a, b) Optical microscopy image of the $WSe_2/WS_2/WSe_2$ heterotrilayer with a twist angle of 3°, with the heterojunctions encapsulated with flakes of h-BN.

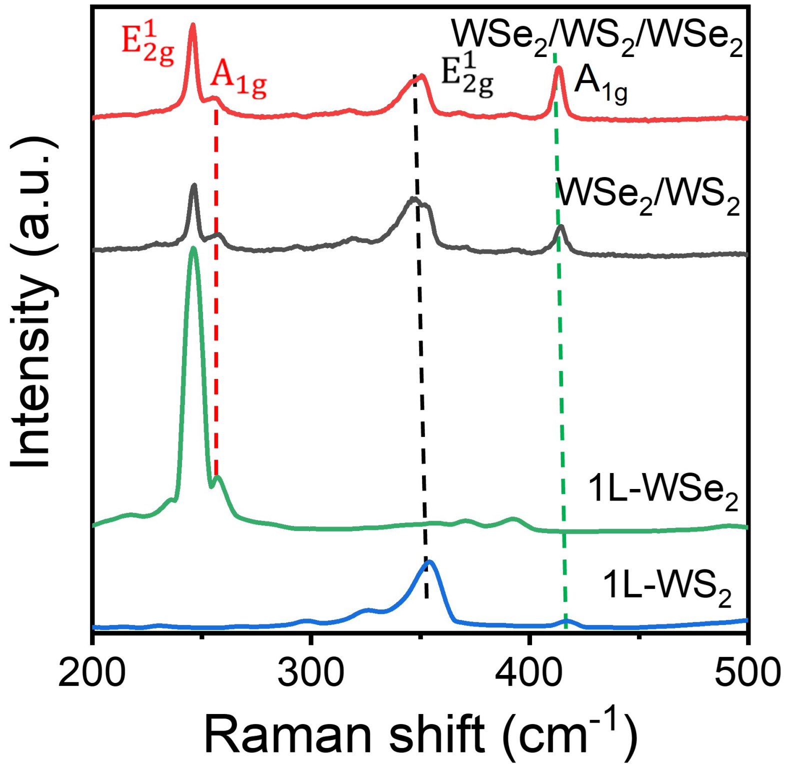

Figure S2 Raman spectra of 1L-WS_2, 1L-WSe_2, WSe_2/WS_2 heterobilayer with a twist angle of 3° and the twisted $WSe_2/WS_2/WSe_2$ heterotrilayer.

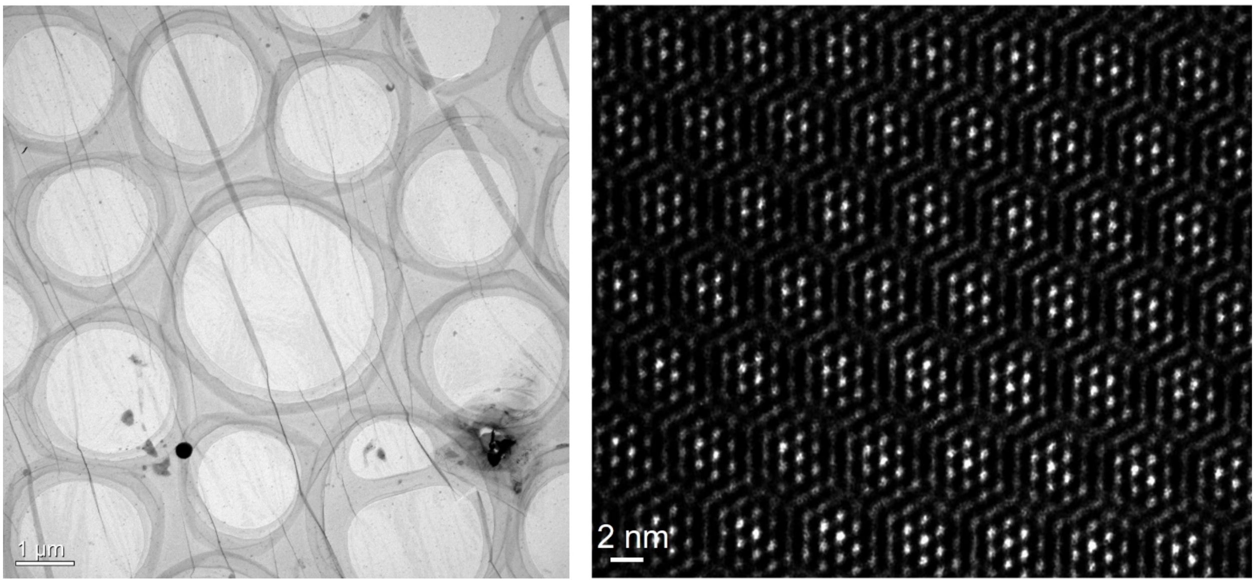

Figure S3 Low-magnification HRTEM image of a twisted-angle heterobilayer. HRTEM images of the heterobilayer with twist angles of 3.5°.

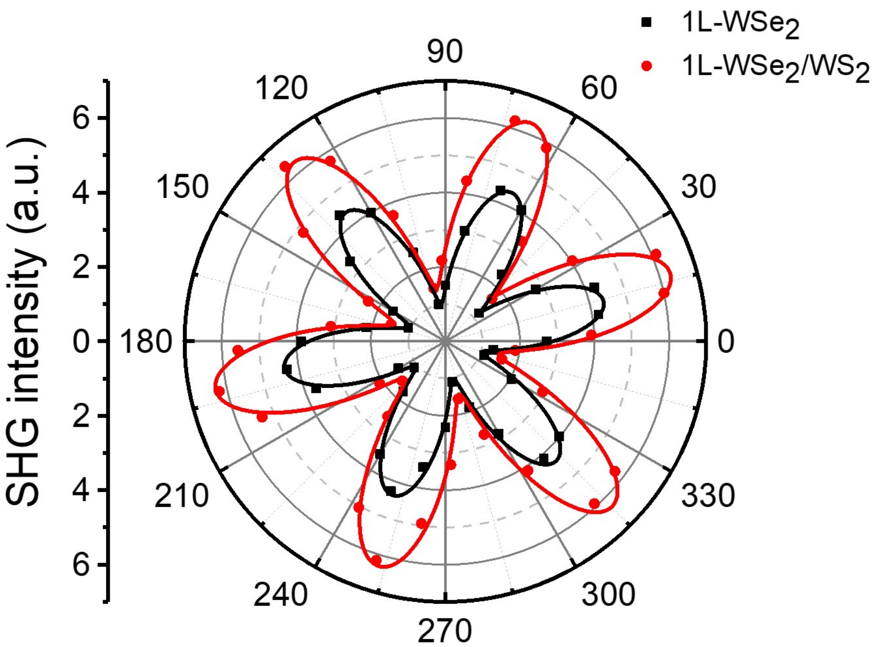

Figure S4 Determination of the twist WSe_2/WS_2 and 1L-WSe_2 by SHG measurement. Sixfold symmetry in the SHG intensity (points) for the 1L-WSe_2 (black) and the twist WSe_2/WS_2 heterobilayer (red).

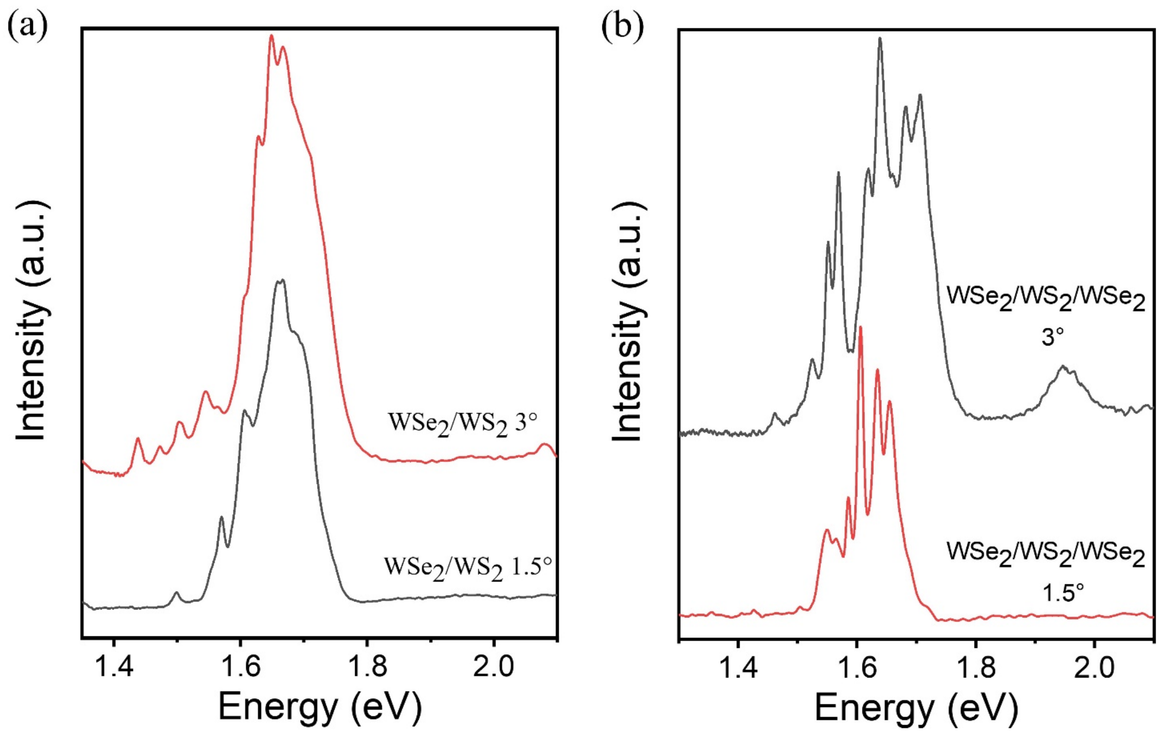

Figure S5 (a) Representative PL spectra of the WSe_2/WS_2 heterobilayer with a twist angle of 3° and 1.5°. PL spectrum of WSe_2/WS_2 heterobilayer at 6 K under an excitation power density of 0.3 mW. (b) The normalized PL spectra of the twisted $WSe_2/WS_2/WSe_2$ heterotrilayer with twist angle of 3° and 1.5° at 6 K. The PL spectrum shows a splitting phenomenon at 6 K under an excitation power density of 0.3 mW.

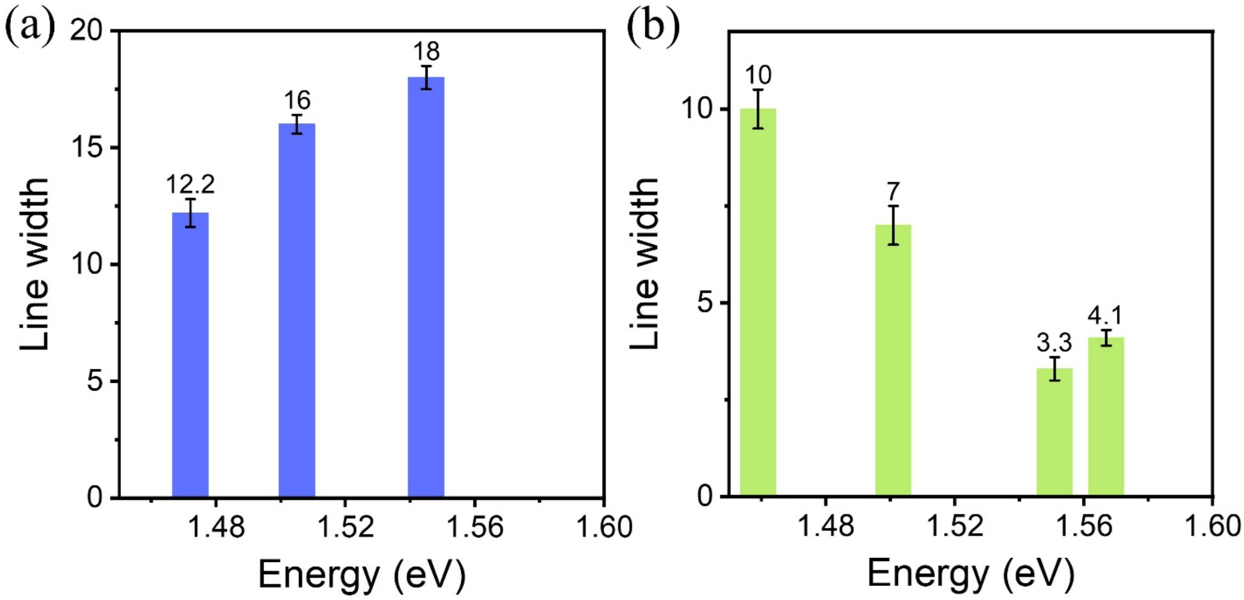

Figure S6 (a, b) Representative PL spectra of WSe_2/WS_2 heterobilayer with a twist angle of 3° and the $WSe_2/WS_2/WSe_2$ heterotrilayer with a twist angle of 3°. Line widths of moiré excitons are obtained for the twisted WSe_2/WS_2 heterobilayer and the $WSe_2/WS_2/WSe_2$ heterotrilayer.

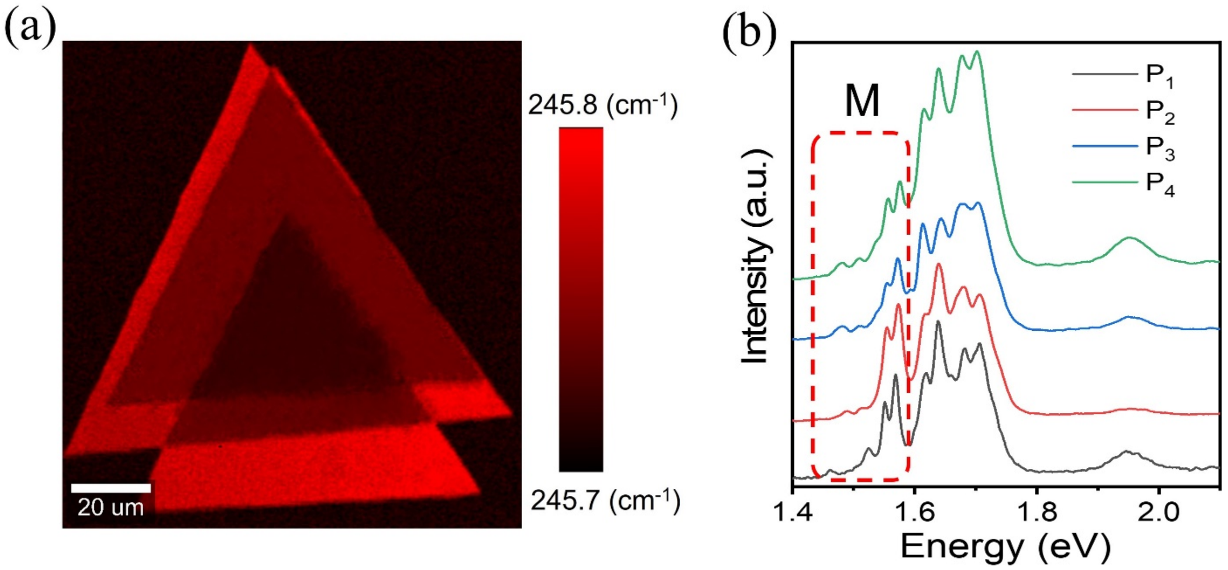

Figure S7 (a) The Raman position mapping of the twisted $WSe_2/WS_2/WSe_2$ heterotrilayer. (b) PL spectra of twisted $WSe_2/WS_2/WSe_2$ heterotrilayer with different positions at 6 K.

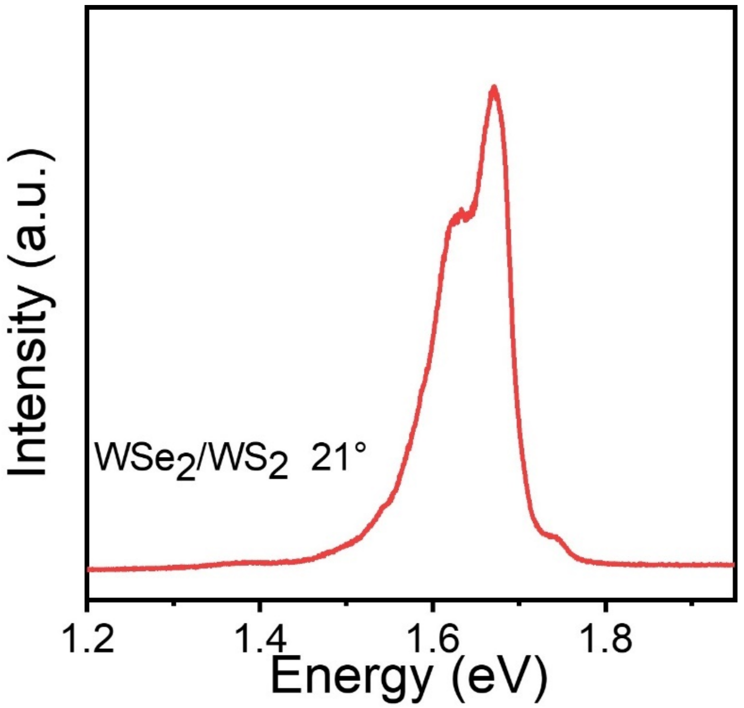

Figure S8 Representative PL spectra of WSe_2/WS_2 heterobilayer with a twist angle of 21° at 6 K.

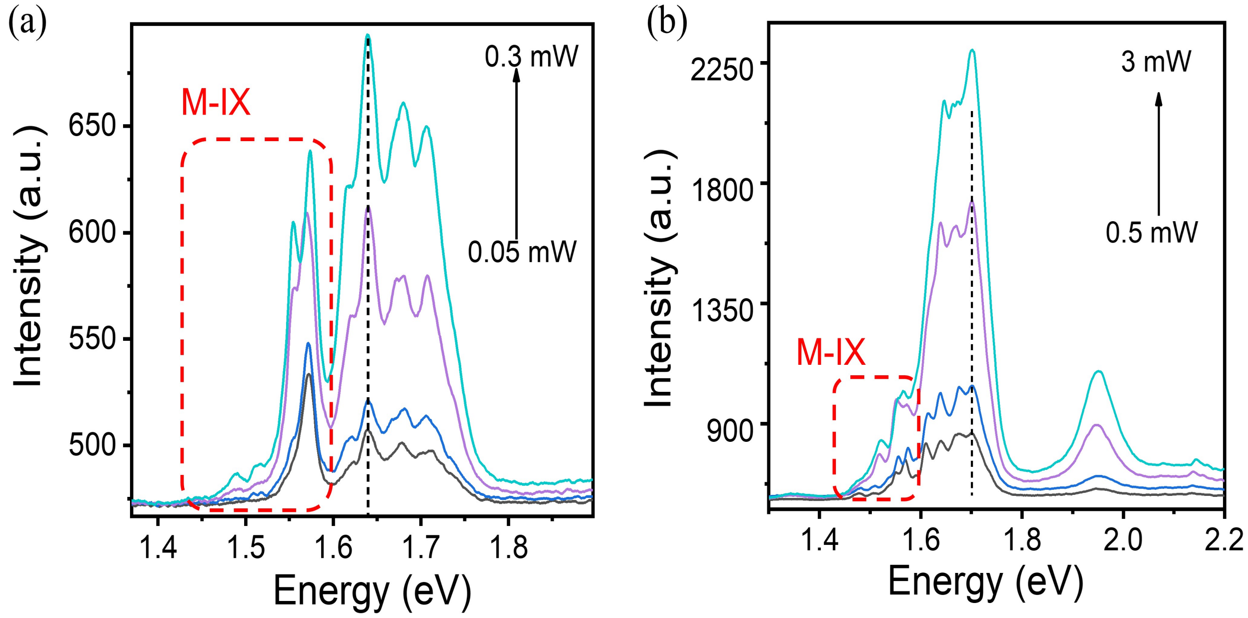

Figure S9 (a, b) The PL spectra of the twisted $WSe_2/WS_2/WSe_2$ heterotrilayer with twist angle of 3° as a function of excitation power under 532 nm laser excitation at 6 K.

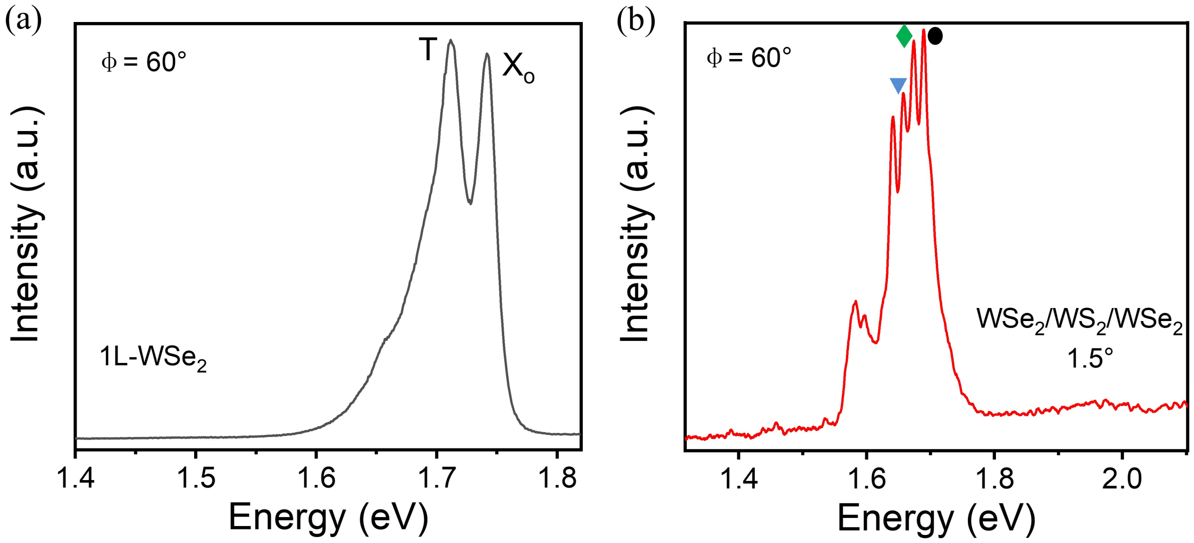

Figure S10 (a) A line cut of Fig. 5(a) at ϕ = 60°, showing multiple intralayer exciton peaks in the monolayer WSe_2. (b) A line cut of Fig. 5(b) at ϕ = 60°, showing multiple emission lines in the twisted $WSe_2/WS_2/WSe_2$ heterotrilayer with a twist angle of 1.5°.

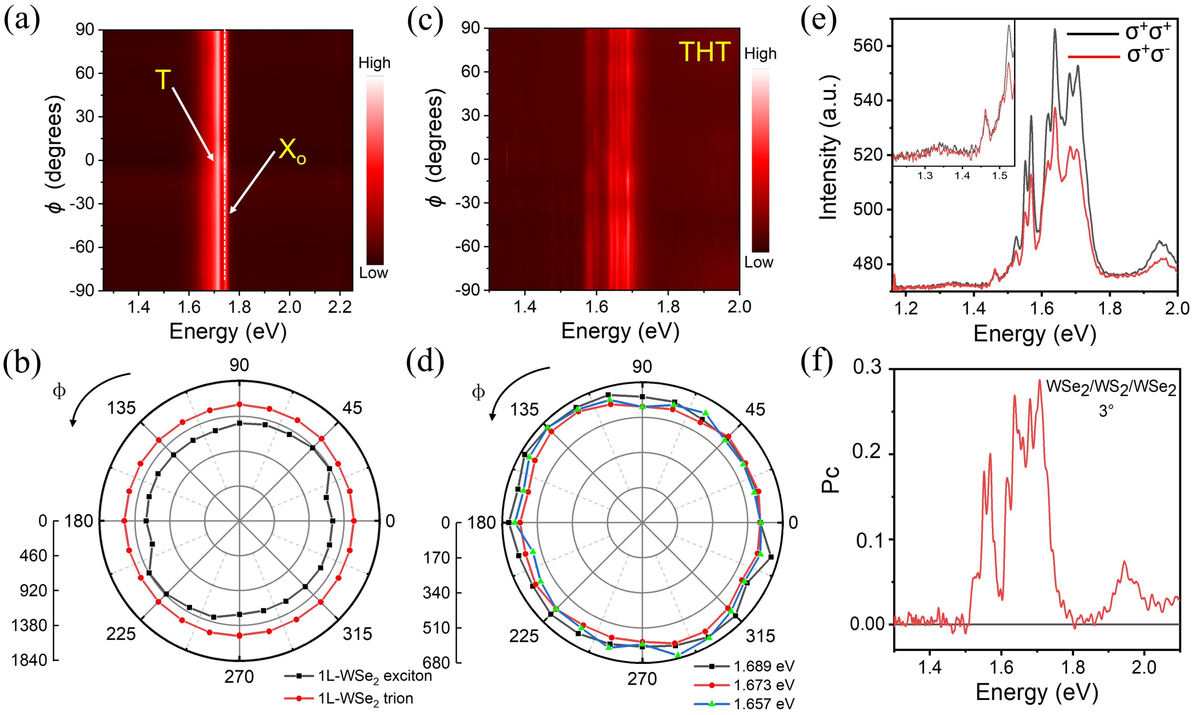

Figure S11 Valley polarization and linear polarization of trapped excitons in $WSe_2/WS_2/WSe_2$ heterotrilayer. (a, b) Line tangent point of exciton energy in the monolayer WSe_2 as a function of the angle ϕ between the half-wave plate and linear polarizer. (c, d) Linear polarization spectra of the twisted $WSe_2/WS_2/WSe_2$ heterotrilayer with a twist angle of 1.5°. (e, f) Circularly polarized photoluminescence spectrum for σ^+ and σ^- excitation of the $WSe_2/WS_2/WSe_2$ heterotrilayer with a twist angle of 3° at 6 K. The relationship between the degree of circular polarization and the emission wavelength are obtained from the spectrum of 5(e).

Table 1 : *g*-factors of both neutral excitons (X_0) and moiré excitons (M_1, M_2)

g-factors	M_1	M_2	X_0
Position 1	-12.1± 0.5	-11.2± 0.2	-6.1± 0.4
Position 2	-11.6± 0.2	-9.8± 0.3	-4.8± 0.5
Position 3	-10.8± 0.5	-10.2± 0.5	-5.1± 0.2

First-principles calculation of the bandgaps of the $WSe_2/WS_2/WSe_2$ heterotrilayer.

(1) Computational method

Our density functional theory (DFT) calculations are performed using the Vienna *ab initio* simulation package (VASP)[1] with the projected augmented wave potential[2]. The generalized gradient approximation developed by Perdew–Burke–Ernzerhof (GGA-PBE) is used to treat the exchange and correlation functional[3]. The DFT-D3 method with Becke-Jonson damping is used to simulate the interlayer van der Waals (vdW) interaction [4, 5]. The valence states $5d^5 6s^1$ for W, $3s^2 3p^4$ for S, and $4s^2 4p^4$ for Se are used with an energy cutoff of 400 eV for the plane wave basis set. A 12×12×1 Γ-centered k-grid is used for 3L-$WSe_2/WS_2/WSe_2$ in bulk-like ABA stacking, while only Γ point is used for 3L-$WSe_2/WS_2/WSe_2$ moiré superlattice. The atomic geometries in both bulk-like and moiré superlattice with a vacuum layer of about 20 Å in the Z direction are fully optimized by relaxing all atoms until the residual force on each atom is less than 0.01 eV/Å and the energy convergence criterion is set at 10^{-4} eV. The electronic band structures are calculated along the high symmetry M- Γ -K-M paths in the Brillouin zone (BZ).

(2) Calculated electronic band structures for 3L-$WSe_2/WS_2/WSe_2$ lattice without twist angle

The structure of 3L-$WSe_2/WS_2/WSe_2$ in bulk-like ABA stacking without twist angle (θ = 0) is given in Supplementary Fig. 12(a). It has a 9-atom hexagonal unit cell in *P-6m2* (D_{3h}^1, No. 187) symmetry with equilibrium lattice parameters a = 3.2087 Å and c = 34 Å, with three W atoms occupying 2*h* (1/3, 2/3, 0.3166)-W_1, and 1*f* (2/3, 1/3, 0.5)-W_2 Wyckoff positions, meanwhile two S and four Se atoms occupying 2*h* (1/3, 2/3, 0.4548)-S, 2*i* (2/3, 1/3, 0.7330)-Se_1, and 2*i* (2/3, 1/3, 0.6337)-Se_2 Wyckoff positions, respectively. The electronic band structure is plotted in Supplementary Fig. 12(b). Our results show that 3L-$WSe_2/WS_2/WSe_2$ in bulk-like ABA stacking is a semiconductor with an indirect band gap of 0.831 eV. *The lowest conduction band width is 1339 meV* and the conduction band minimum (CBM) is located at K points; on the other hand, *the highest valence band width is 1048 meV* and the valence band maximum (VBM) is located at the Γ point. However, the direct band gap of 0.980 eV at K point is about 0.15 eV larger than the indirect band gap of 0.831 eV, revealing that the 3L-$WSe_2/WS_2/WSe_2$ in bulk-like ABA stacking without twist angle (θ = 0) is an indirect semiconductor. From the partial density of states (DOSs) [Supplementary Fig. 12(c)], we can see that the states around the VBM at Γ point are almost contributed by W_2-5*d* orbitals and partial W_1-

5*d* orbitals, while the states around the CBM-*K* are almost contributed by W_2-5*d* orbitals [Supplementary Fig. 12(c)]. The contributions from other orbitals such as S-3*p* and Se-4*p* are very small.

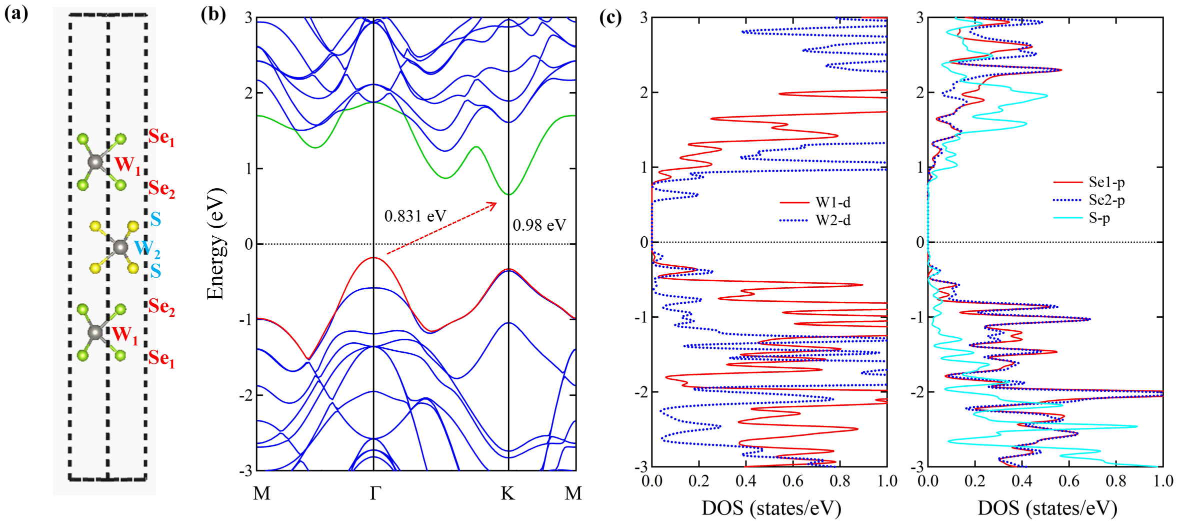

Figure S12 (a) The structure of 3L-$WSe_2/WS_2/WSe_2$ in bulk-like ABA stacking without twist angle ($\theta = 0$) in *P-6m2* (D_{3h}^1, No. 187) symmetry. The Wyckoff positions for W, S and Se atoms are listed in text. (b) Electronic band structure for 3L-$WSe_2/WS_2/WSe_2$ in bulk-like ABA stacking without twist angle under GGA-PBE. The Fermi level is set to zero eV. The valence band maximum (VBM) and conduction band minimum (CBM) are at Γ and K point, respectively. (c) The partial DOSs of W_1-5*d*, W_2-5*d*, S-3*p*, Se_1-4*p*, and Se_2-4*p* electrons.

(3) Electronic band structures for $WSe_2/WS_2/WSe_2$ moiré superlattice with a twist angle of 3.15°

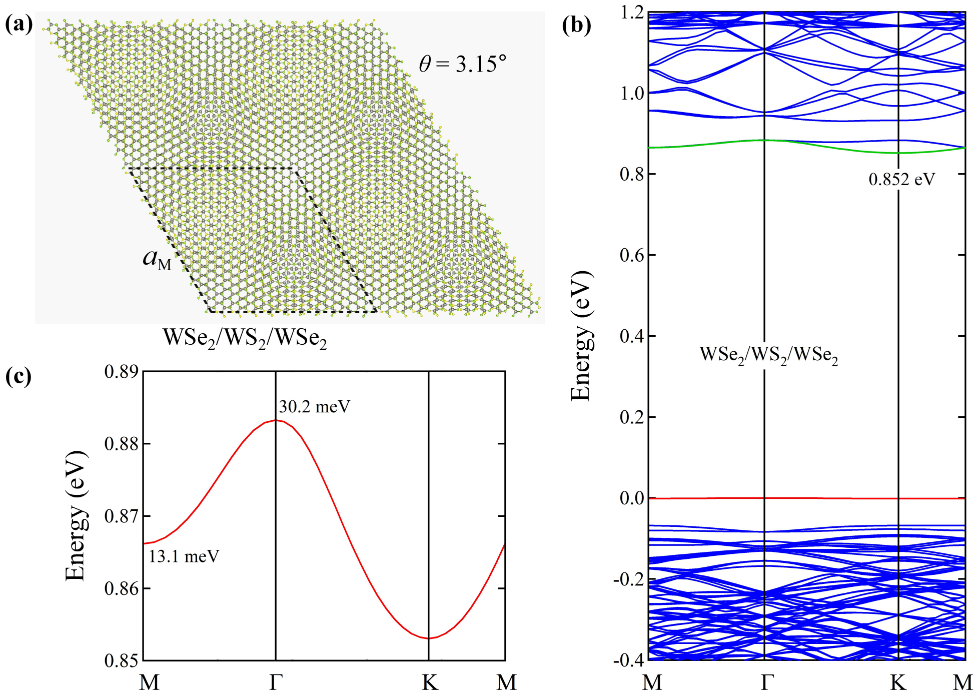

Figure S13 (a) Top view of 3L $WSe_2/WS_2/WSe_2$ moiré superlattice with a twist angle of 3.15° in *P-6* (D_{3h}^1, No. 174) symmetry. (b) Electronic band structure of 3L $WSe_2/WS_2/WSe_2$ moiré superlattice under GGA-PBE. The Fermi level is set to zero eV relative to the valence band maximum at Γ point. The conduction band minimum is at K point with a direct band gap of 0.851 eV at K point. (c) The direct band gap along the high symmetry M-Γ-K-M paths in BZ, estimated as the difference between the lowest conduction band (green line) and highest valence band (red line). The change of the direct band gap relative to K point are 13.1 meV at M and 30.2 meV at Γ, respectively.

To get a better understanding on the electronic behavior of 3L $WSe_2/WS_2/WSe_2$ moiré superlattice (MSL), a MSL with a twist angle of 3.15° is calculated and plotted in Supplementary Fig. 13(a). This moiré superlattice has 993 W, 662 S, and 1324 Se atoms in a hexagonal unit cell in *P-6* (D_{3h}^1, No. 174) symmetry with equilibrium lattice parameters a_M = 58.387 Å and c = 34 Å [Supplementary Fig. 13(a)]. The calculated electronic band structure is plotted in Supplementary Fig. 13(b). The conduction band minimum (CBM) is located at K point; while the valence band maximum (VBM) is located at the Γ point. However, the highest valence band width is only 1 meV, showing a flat valence band behavior in 3L $WSe_2/WS_2/WSe_2$ moiré superlattice. Thus, the 3L $WSe_2/WS_2/WSe_2$ MSL is a semiconductor with a direct band gap of 0.851 eV [Supplementary Fig. 13(b)] at K point, which is larger than but close to the indirect band gap of 0.831 eV for 3L-$WSe_2/WS_2/WSe_2$ in bulk-like ABA stacking without

twist angle [Supplementary Fig. 12(b)].

On the other hand, the lowest conduction band width is estimated to be 30.2 meV, which is clearly smaller than the lowest conduction bandwidth of *1339* meV for 3L-$WSe_2/WS_2/WSe_2$ in bulk-like ABA stacking without twist angle (θ = 0) [Supplementary Fig. 12(b)]. To get better understanding on the electronic behavior of $WSe_2/WS_2/WSe_2$ moiré superlattice, we have also plotted the direct band gap in Supplementary Fig. 13(c) along the high symmetry M-Γ-K-M paths in the BZ, estimated as the difference between the lowest conduction band and highest valence band at each *k*-point. The calculated *minimum* direct band gap is 0.851 eV at K-point, which is about 1 meV samller than but very close to the indirect band gap of 0.852 eV, revealing that $WSe_2/WS_2/WSe_2$ moiré superlattice is a semiconductor with *quasi*-direct band gap of 0.851 eV. The change of the direct band gap relative to *K* point are 13.1 meV at M and 30.2 meV at Γ, respectively. These calculated results give a good understanding of the splitting peak spacing of moiré excitons observed in our experiments.

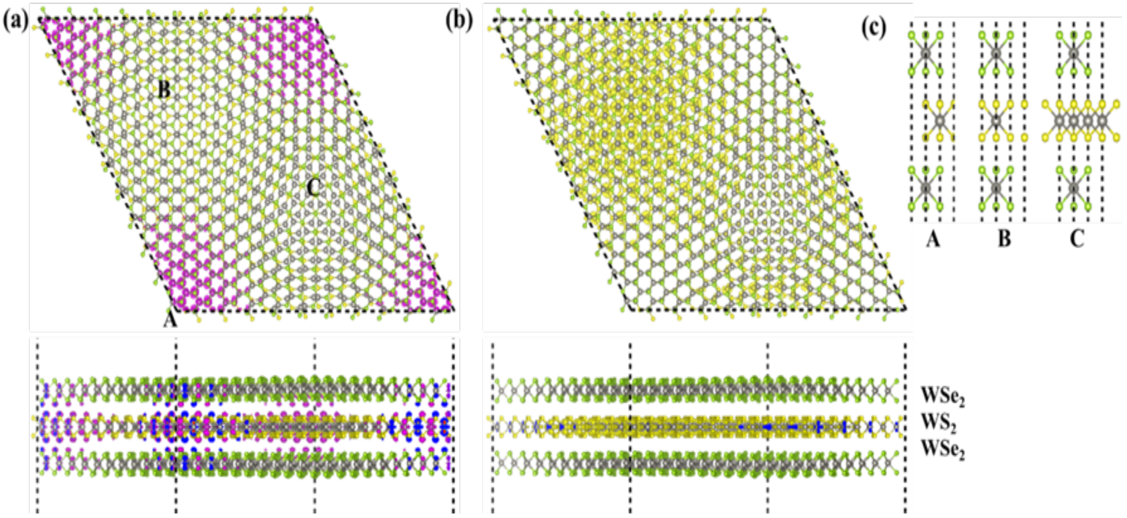

Figure S14 The band-decomposed partial charge densities of 3L $WSe_2/WS_2/WSe_2$ moiré superlattice with a twist angle of 3.15° at VBM-Γ point (a) and CBM-K point (b). The isosurfaces are 0.0001 *e*/Bohr^3. (c) The atomic configurations at high symmetric points A, B and C.

Following the ref's comments, we have further calculated the band-decomposed partial charge densities and plotted it in Figure 14. It is shown that the conduction-band minimum states localized around the high symmetric point B and almost contributed by W-5d orbitals in WS_2 middle layer. However, we cannot extract from the distribution of the Kohn-Sham orbitals information about the spatial variation of the moire potential, because the orbitals are calculated in the k-space.

References

[1] Kresse, G.; Furthmüller, J., Efficient iterative schemes for ab initio total-energy calculations using a plane-wave basis set. *Physical review. B, Condensed matter* **54**, 11169-11186 (1996).

[2] Blöchl, P. E., Projector augmented-wave method. *Physical Review B* **50**, 17953-17979 (**1994**).

[3] Perdew, J. P.; Burke, K.; Ernzerhof, M., Generalized Gradient Approximation Made Simple. *Physical Review Letters* **77**, 3865-3868 (1996).

[4] Grimme, S.; Antony, J.; Ehrlich, S.; Krieg, H., A consistent and accurate *ab initio* parametrization of density functional dispersion correction (DFT-D) for the 94 elements H-Pu. *The Journal of Chemical Physics* 132, 154104 (2010).

[5] Grimme, S.; Ehrlich, S.; Goerigk, L., Effect of the damping function in dispersion corrected density functional theory. *Journal of Computational Chemistry* **32**, 1456-1465 (2011).
